# Intraoperative dexmedetomidine on postoperative pain in gastrointestinal surgery: an observational study

**DOI:** 10.1097/JS9.0000000000000360

**Published:** 2023-03-31

**Authors:** Xuecai Lv, Haoyun Zhang, Jie Gao, Aisheng Hou, Yulong Ma, Zhikang Zhou, Weidong Mi, Hong Zhang, Yanhong Liu

**Affiliations:** a Department of Anesthesiology, First Medical Center of Chinese PLA General Hospital; b Chinese PLA Medical School; c National Clinical Research Center for Geriatric Diseases, PLA General Hospital, 28 Fuxing Road, Haidian District, Beijing China

**Keywords:** anxiety, dexmedetomidine, gastrointestinal surgery, patient satisfaction, postoperative pain, propensity score matching

## Abstract

Dexmedetomidine (DEX) is widely used for adjuvant sedation and analgesia in gastrointestinal surgeries. The authors aimed to reassess the effects of intraoperative DEX on acute pain by comprehensive analysis of the multiple dimensions of pain.

**Materials and Methods:**

In this multicentre cohort study, patients undergoing gastrointestinal surgeries were prospectively enrolled in the China Acute Postoperative Pain Study. Patients were divided into DEX and non-DEX groups based on whether DEX was used during surgery. Patient satisfaction with pain treatment (rated on a numeric rating score, 0–10) and other pain-related outcomes were evaluated using the International Pain Outcome Questionnaire on the first postoperative day. The effects of intraoperative DEX were analyzed using logistic or linear regression for dichotomous or continuous variables, respectively. Propensity score matching and subgroup analyses were performed to appraise the correlation between intraoperative DEX and postoperative pain outcomes.

**Results:**

Of the 1260 patients eligible for analysis, 711 (56.4%) received intraoperative DEX. Propensity score matching resulted in 415 patients in each group. Intraoperative DEX was associated with higher patient satisfaction (β: 0.556; 95% CI: 0.366–0.745), and a decrease in the percentage of time spent in severe pain (β: −0.081; 95% CI: −0.104– −0.058), anxiety (odds ratio: 0.394; 95% CI: 0.307–0.506), helplessness (odds ratio: 0.539; 95% CI: 0.411–0.707), and postoperative opioid consumption (β: −16.342; 95% CI: −27.528– −5.155).

**Conclusions:**

Intraoperative DEX was associated with the prognosis of acute postoperative pain in multiple aspects in patients undergoing major gastrointestinal surgery, including increased patient satisfaction, and a reduction in the duration of severe pain, postoperative anxiety and helplessness, and postoperative opioid consumption. Future studies to determine the dose and timing of DEX administration on pain-related outcomes are warranted.

## Introduction

Highlights

- Acute postoperative pain after gastrointestinal surgery is challenging.
- Intraoperative use of dexmedetomidine (DEX) is becoming increasingly common.
- The outcome of acute postoperative pain was analyzed in multiple dimensions in this cohort study.
- Intraoperative DEX was related to better patient satisfaction and less postoperative anxiety.
- Intraoperative DEX was associated with shorter duration of severe pain and less opioid consumption.

Over 240 million surgical procedures are performed annually worldwide^1^, and acute postoperative pain is insufficiently treated, with more than 50% of patients experiencing moderate to severe pain after surgery^2^. In gastrointestinal surgeries, ~30% of patients experience severe pain with a significant impact on their mood and sleep after surgeries, and 12% of patients visit the emergency department for pain-related symptoms^3^. Uncontrolled acute postoperative pain is related to the development of persistent pain after surgery, which is associated with increased gastrointestinal symptoms and a decreased overall quality of life^4^. Furthermore, opioid-related side effects are of great concern in these patients^5^. The side effects of opioids, such as nausea, vomiting, gastrointestinal dysfunction, drowsiness, and respiratory depression, may outweigh the analgesic benefit, as they may impair postoperative rehabilitation^6^. The gastrointestinal side effects of opioids are particularly undesirable after abdominal surgery^7^.

Dexmedetomidine (DEX) is a selective ɑ_2_ receptor agonist with sedative, anxiolytic, and antihypertensive properties^8^,^9^. Several randomized controlled trials have focused on the effect of intraoperative DEX on acute postoperative pain in abdominal surgeries^10^,^11^. The results have shown that DEX administered during abdominal surgeries reduces the postoperative opioid requirement for pain treatment on the first postoperative day. In one study, intraoperative DEX combined with lidocaine infusion reduced the intensity of pain on postoperative day one^12^. In other studies, however, no significant reduction in pain intensity was noticed^13^,^14^.

The experience of postoperative pain can include several dimensions: sensory (intensity and character of pain), affective (emotional component), and impact (ability to function). All these items should be evaluated to fully understand the control of acute pain. However, in previous studies, less attention has been paid to the comprehensive evaluation of the effect of DEX on all aspects of pain. Therefore, in this exploratory study, we hypothesized that intraoperative DEX is associated with multiple aspects of acute postoperative pain, including postoperative patient satisfaction with pain treatment, pain intensity, pain interference with emotional and physical functions, postoperative opioid consumption, and adverse events of pain treatment. We tested this hypothesis in gastrointestinal surgery patients using data collected in the China Acute Postoperative Pain Study.

## Materials and methods

### Study design

This study was reported in line with the strengthening the reporting of cohort, cross-sectional and case-control studies in surgery (STROCSS) criteria^15^, Supplemental Digital Content 1, http://links.lww.com/JS9/A226. In this cohort study, we analyzed data collected in the China Acute Postoperative Pain Study (CAPOPS, https://mazuidata.medbit.cn/). CAPOPS is a national quality improvement and registry project that aims to improve the treatment of patients with postoperative pain in China; it was established largely drawing on the experiences of the PAIN-OUT project^16^–^18^. CAPOPS was registered at http://www.chictr.org.cn (ChiCTR1900025237) on 17 August 2019 and initiated in September 2019. The CAPOPS registry enrolled surgical patients from 122 centers in China between September 2019 and August 2021. Among the 122 participating centers, there were 115 (94.3%) tier 3 hospitals (tertiary hospitals), and 7 (5.7%) were tier 2 hospitals. Research approval was obtained from each participating center. Written or oral consent was obtained from each participant according to the requirements of the local ethics committee. The protocol for the current analysis was submitted and reviewed by the CAPOPS committee prior to data access.

The web-based database of CAPOPS was open to investigators at participating centers who signed an agreement and received training on the standard operating procedure. Adult patients on their first postoperative day who agreed to participate were required to complete the Chinese version of the International Pain Outcome Questionnaire (IPOQ, Supplementary 1, Supplemental Digital Content 2, http://links.lww.com/JS9/A227)^19^. The research assistant reviewed the medical records and filled in the process questionnaire, which included patient demographics, perioperative pain treatment, and surgical information. Data were entered online at the participating centers and reviewed by the central center to perform additional data quality checks. In cases of missing data or irregularities, an inquiry was made.

### Population

Adult patients (age≥18 years) who underwent general anesthesia (GA) for major gastrointestinal surgeries, including gastrectomy (partial or total gastrectomy) and enterectomy (resection of the small intestine, colon, and/or rectum), were considered for inclusion in the current study. Patients who met the inclusion criteria were screened in the CAPOPS database. Patients were excluded from analysis if they received regional anesthesia (RA) alone during the surgery, underwent another surgery during the same hospitalization period, or their reported pain outcomes in the IPOQ were missing.

The patients who received intravenous DEX during the operation formed the DEX group, while those who did not receive intravenous DEX formed the non-DEX group.

### Outcomes

The primary outcome was patient satisfaction with pain treatment rated on a numeric rating score [(NRS), 0=extremely dissatisfied, 10=extremely satisfied]. This score was built in as the results for the question ‘How satisfied you are with the results of your pain treatment since your surgery?’ The secondary outcomes included: pain severity, including the worst and least pain scores since surgery (rated on NRS, 0=no pain, 10=maximum pain), incidence of severe pain (NRS of the worst pain≥7), and percentage of time spent in severe pain (0–100%); pain interference with emotional and physical functions, including anxiety, helplessness, performing activities in bed, taking a deep breath, coughing, and sleep. An NRS score greater than 0 indicated that pain had an effect on certain functions; postoperative opioid consumption and adverse events of pain treatment. Morphine equivalent doses were calculated for comparison of opioid consumption^20^–^22^. Adverse events of pain treatment such as nausea, drowsiness, pruritus, and dizziness were analyzed. An NRS score greater than 0 indicated that pain treatment had adverse events; and perception of care, that is the wish for more treatment (yes/no).

### Confounders

Confounders included demographics (age, sex, BMI, comorbidity, and history of preoperative chronic pain), perioperative medication (intraoperative analgesics, postoperative analgesics, and the frequency of postoperative DEX use, including use of DEX in recovery rooms and/or wards), as well as surgical and anesthetic parameters (operation time, location of surgery, type of surgery, and anesthesia methods). The types of procedures were classified into gastrectomy (partial or total gastrectomy) and enterectomy (resection of the small intestine, colon, and/or rectum), according to the location of the operation. Procedures were divided into minimally invasive and open procedures according to their surgical approaches. Minimally invasive procedures included laparoscopic and robot-assisted procedures. All surgical types were screened according to the International Classification of Diseases, Ninth Revision, Clinical Modification (ICD-9-CM) codes. Anesthesia methods were classified into GA alone and GA combined with RA (GA+RA) according to whether RA was used. A detailed list of the specific RA techniques is shown in Table S1, Supplemental Digital Content 3, http://links.lww.com/JS9/A228.

### Statistical analysis

Statistical analysis was performed using R (version 3.5.3; R Foundation for Statistical Computing) and SPSS software (version 26.0; SPSS). Continuous variables that followed a normal distribution are presented as mean and SD, while variables that followed a non-normal distribution are presented as median and interquartile range (IQR). The normality of the distribution was assessed using histograms and the Shapiro–Wilk test. Categorical variables are presented as frequency and percentage. Continuous variables were assessed by an independent *t*-test or a Mann–Whitney *U* test. Categorical variables were analyzed by the *χ* ^2^-test.

The sample size was based on the number of eligible patients in the CAPOPS cohort. Moreover, a sample size estimate based on a difference of 0.5 in patient satisfaction scores between groups, an SD of (2)2, a two-sided α of 0.05, and a power of 80% showed that 253 cases in each group would be sufficient^23^,^24^. Therefore, we considered the sample size adequate.

Linear regression models were used to assess the effect of intraoperative DEX on patient satisfaction, worst pain score, least pain score, percentage of time spent in severe pain, and postoperative consumption of opioids. The results of linear regression were reported as β value, that is, the regression coefficient. We applied a multivariate logistic regression approach to evaluate the clinical effect of intraoperative DEX on binary outcomes, including severe pain, anxiety, helplessness, moving in bed, deep breath or coughing, sleep abnormalities, adverse events of pain treatment, and patients’ wish for more treatment. The results of the logistic regression were reported using odds ratio. The receiver operating characteristic curves were plotted to demonstrate the performance of the multivariate regression models, where continuous outcomes were converted to dichotomous outcomes based on the median.

Propensity score matching (PSM) was applied to address the potential residual confounding effect of the covariates. The propensity score, a composite score, was computed based on the probability of patients receiving intravenous DEX and derived from synthesized baseline parameters using the multivariate logistic regression model^25^. After propensity score generation, patients who received intraoperative DEX were matched to those who did not receive intraoperative DEX at a 1 : 1 ratio using greedy propensity score techniques and a calliper within 0.02 SD of the propensity score distribution^26^. Standardized mean differences for all covariates were calculated before and after matching, with 10% or more considered as indicative of imbalance.

A prespecified subgroup analysis was performed to appraise the primary correlation between intraoperative DEX and postoperative pain according to age, sex, BMI, comorbidity, duration of surgery, type of surgery, and anesthesia methods^27^. Exploratory analyses were performed to assess the association of the intraoperative DEX dose with postoperative patient satisfaction and other pain-related outcomes using linear regression or logistic regression according to the character of the data. To reduce the chances of obtaining false-positive results (type I errors) in multiple comparisons, we used Holm’s procedure to correct *P* values^28^. A two-sided *P* value of less than 0.05 was considered statistically significant.

## Results

**Figure 1:**
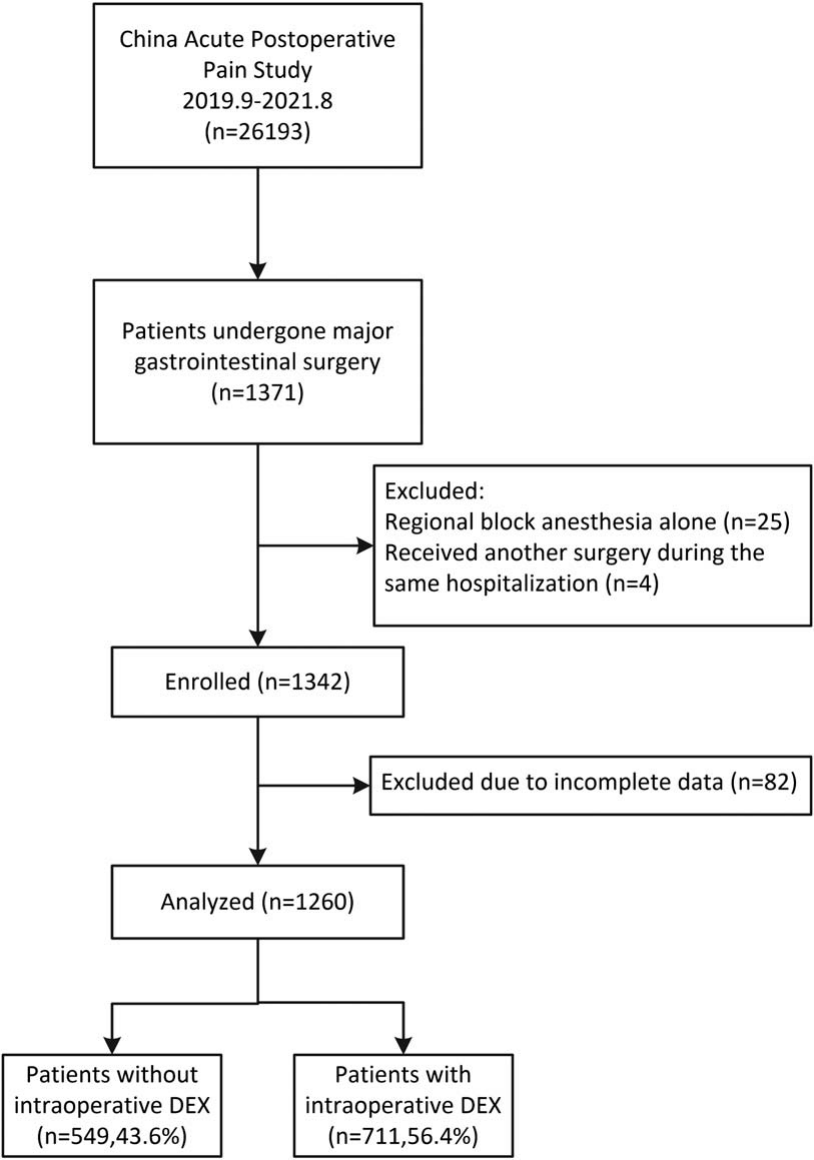
Analysis flow chart. DEX, dexmedetomidine; CAPOPS, China Acute Postoperative Pain Study.

A total of 26 193 cases were enrolled in CAPOPS from September 2019 to August 2021. Of these, 1371 patients underwent major gastrointestinal surgeries. Among the enrolled patients, 25 cases were excluded because the patients received RA alone, four cases were excluded because they underwent another surgery during the same hospitalization period, and 82 cases were excluded due to patient reported pain outcomes partially missing, including the least pain score (one case), the percentage of time spent in severe pain (one case), pain interference with physical functions (two cases), adverse events of pain treatment (three cases), wish for more treatment (71 cases), and patient satisfaction (four cases). After applying the inclusion and exclusion criteria, 1260 eligible patients from 63 centers remained in the analysis, with 711 (56.4%) in the DEX group and 549 (43.6%) in the non-DEX group (Fig. 1). The baseline characteristics of the patients with or without intraoperative DEX are summarized in Table 1. DEX was administered in patients with more comorbidities, longer operation time, and undergoing more invasive procedures. More patients in the DEX group received combined GA and RA than those in the non-DEX group. Fewer patients in the DEX group received intraoperative and postoperative nonsteroidal anti-inflammatory drugs and postoperative DEX than those in the non-DEX group. The average follow-up time of patients was 20.5 ± 2.7 h with no difference between the two groups.

**Table 1:** Characteristics of the primary cohort before and after matching.

	Unmatched patients				Propensity-score-matched patients			
Parameter	Non-DEX group (*n*=549)	DEX group (*n*=711)	*P*	SMD	Non-DEX group (*n*=415)	DEX group (*n*=415)	*P*	SMD
**Patient characteristics**								
Age, year	63.0 (54.0–69.5)	63.0 (53.0–69.0)	0.532	0.035	62.0 (54.0–69.0)	63.0 (53.0–70.0)	0.567	0.042
Male sex, *n* (%)	325 (59,2)	451 (63.4)	0.141	0.087	260 (62.7)	248 (59.8)	0.433	0.059
BMI, kg/m^2^	23.5 (21.3–26.0)	23.6 (21.2, 25.9)	0.697	0.010	23.6 (21.3–26.0)	23.4 (21.0–25.5)	0.231	0.092
Comorbidity, *n* (%)	220 (40.1)	376 (52.9)	<0.001	0.259	193 (46.5)	195 (47.0)	0.945	0.010
Hypertension	115 (20.9)	216 (30.4)	<0.001	0.217	99 (23.9)	110 (26.5)	0.424	0.061
Diabetes	48 (8.7)	75 (10.5)	0.330	0.061	42 (10.1)	42 (10.1)	1.000	<0.001
Affective disorders	0 (0)	3 (0.4)	0.347	0.092	0 (0)	0 (0)	1.000	<0.001
Tumor	56 (10.2)	98 (13.8)	0.066	0.110	53 (12.8)	53 (12.8)	1.000	<0.001
Current smoker	1 (0.2)	6 (0.8)	0.236	0.093	1 (0.2)	1 (0.2)	1.000	<0.001
Preoperative chronic pain, *n* (%)	19 (3.5)	36 (5.1)	0.214	0.079	18 (4.3)	22 (5.3)	0.627	0.045
**Perioperative medication**								
Intraoperative analgesics								
Consumption of opioids, mg	135.0 (58.4–200.0)	147.0 (105.0–230.0)	0.002	0.148	135.0 (60.0–205.0)	140.0 (82.0–225.5)	0.250	0.022
NSAIDs, *n* (%)	259 (47.2)	435 (38.8)	0.003	0.169	175 (42.2)	192 (46.3)	0.263	0.083
Postoperative analgesics								
NSAIDs, *n* (%)	69 (12.6)	52 (7.3)	0.002	0.176	39 (9.4)	35 (8.4)	0.715	0.034
Local anesthetics, *n* (%)	4 (0.7)	2 (0.3)	0.413	0.063	4 (1.0)	2 (0.5)	0.682	0.057
Postoperative use of DEX, *n* (%)	174 (31.7)	121 (17.0)	<0.001	0.347	94 (22.7)	97 (23.4)	0.869	0.017
**Surgical/Anesthetic parameters**								
Duration of surgery, min	156.8 (122.7–204.3)	165.5 (130.6–210.6)	0.020	0.139	158.3 (122.7–205.8)	162.3 (126.7–207.4)	0.767	0.031
Type of surgical , *n* (%)			0.002	0.183			0.879	0.011
Open	134 (24.4)	232 (32.6)			121 (29.2)	123 (29.6)		
Minimally invasive	415 (75.6)	479 (67.4)			294 (70.8)	292 (70.4)		
Location of surgery, *n* (%)			<0.001	0.235			0.440	0.054
Gastrectomy	203 (37.0)	345 (48.5)			170 (41.0)	181 (43.6)		
Enterectomy	346 (63.0)	366 (51.5)			245 (59.0)	234 (56.4)		
Anesthesia methods, *n* (%)			0.016	0.140			0.832	0.015
GA	333 (60.7)	382 (53.7)			248 (59.8)	245 (59.0)		
GA+RA	216 (39.3)	329 (46.3)			167 (40.2)	170 (41.0)		

The data are shown as median (interquartile range) or *n* (%).

Affective disorders including depression, anxiety, phobia, post-traumatic stress disorder (PTSD), and bipolar disorder.

DEX, dexmedetomidine; GA, general anesthesia; NSAID, nonsteroidal anti-inflammatory drug; RA, regional anesthesia; SMD, standardized mean differences.

### Primary analysis

Univariate linear regression analysis revealed significant associations between intraoperative DEX and postoperative patient satisfaction with pain treatment (β: 0.476; 95% CI: 0.289–0.662, *P*<0.001) (Supplementary 2, Table S2, Supplemental Digital Content 3, http://links.lww.com/JS9/A228). A similar result was obtained in the multivariate linear regression analysis (β: 0.556; 95% CI: 0.366–0.745, *P*<0.001) (Table 2).

**Table 2:** Comparison of outcomes before and after propensity score matching.

	Before matching				After matching			
Outcomes	Non-DEX group, (*n*=549)	DEX group, (*n*=711)	OR/β(95% CI)	Adjusted *P*	Non-DEX group, (*n*=415)	DEX group, (*n*=415)	OR/β(95% CI)	Adjusted *P*
**Primary outcome**								
Satisfaction, median(IQR)^a^	9 (8–10)	10 (9–10)	0.556 (0.366,0.745)	<0.001	9 (8–10)	10 (8–10)	0.472 (0.233,0.711)	0.002
**Secondary outcomes**								
Pain intensity								
Worst pain score, median(IQR)^a^	3 (2–5)	4 (3–5)	0.121 (−0.110,0.352)	0.913	3 (2–5)	4 (3–5)	0.205 (−0.074,0.484)	0.751
Least pain score, median(IQR)^a^	1 (0–2)	0 (0–1)	−0.451 (−0.595,−0.306)	<0.001	1 (0–2)	0 (0–1)	−0.357 (−0.538,−0.177)	0.002
Time spent in severe pain, %, median(IQR)^a^	10 (0–30)	10 (0–20)	−0.081 (−0.104,−0.058)	<0.001	20 (0–30)	10 (0–20)	−0.066 (−0.094,−0.038)	<0.001
Severe pain, n (%)^b^	57 (10.4)	77 (10.8)	0.860 (0.586,1.263)	0.913	48 (11.6)	43 (10.4)	0.869 (0.556,1.360)	1.000
Emotional impairment and ability to function								
Anxiety, n (%)^b^	260 (47.4)	208 (29.3)	0.394 (0.307,0.506)	<0.001	197 (47.5)	119 (28.7)	0.434 (0.323,0.582)	<0.001
Helplessness, n (%)^b^	163 (29.7)	144 (20.3)	0.539 (0.411,0.707)	<0.001	122 (29.4)	84 (20.2)	0.598 (0.433,0.826)	0.018
Moving in bed, *n* (%)^b^	501 (91.3)	639 (89.9)	0.867 (0.581,1.293)	0.913	378 (91.1)	377 (90.8)	0.919 (0.566,1.491)	1.000
Deep breathing/coughing, *n* (%)^b^	477 (86.9)	574 (80.7)	0.658 (0.478,0.906)	0.063	361 (87.0)	336 (81.0)	0.617 (0.422,0.903)	0.090
Sleep, *n* (%)^b^	281 (51.2)	365 (51.3)	0.823 (0.648,1.045)	0.441	219 (52.8)	199 (48.0)	0.834 (0.628,1.109)	0.850
Postoperative opioid and adverse events								
Postoperative consumption of opioids, mg, median(IQR)^a^	50.0 (20.0–115.0)	60.0 (20.0–90.0)	−16.342 (−27.528,−5.155)	0.034	65.0 (26.2–119.1)	55.9 (20.0–91.7)	−20.271 (−32.792,−7.750)	0.017
Nausea, *n* (%)^b^	263 (47.9)	240 (33.8)	0.627 (0.492,0.800)	0.002	186 (44.8)	144 (34.7)	0.631 (0.473,0.842)	0.018
Drowsiness, *n* (%)^b^	207 (37.7)	175 (24.6)	0.504 (0.387,0.658)	<0.001	154 (37.1)	108 (26.0)	0.584 (0.427,0.799)	0.009
Pruritus, *n* (%)^b^	54 (9.8)	34 (4.8)	0.473 (0.295,0.759)	0.017	43 (10.4)	19 (4.6)	0.425 (0.240,0.751)	0.026
Dizziness, *n* (%)^b^	216 (39.3)	236 (33.2)	0.751 (0.583,0.968)	0.134	155 (37.3)	135 (32.5)	0.785 (0.581,1.061)	0.693
Perception of care								
Wish for more treatment, *n* (%)^b^	202 (36.8)	139 (19.5)	0.330 (0.250,0.436)	<0.001	149 (35.9)	77 (18.6)	0.393 (0.282,0.547)	<0.001

b Multivariate logistic model adjusted for age, sex, comorbidities, preoperative chronic pain, operation time, location of surgery, anesthesia methods, intraoperative consumption of opioids, intraoperative use of NSAIDs, postoperative use of NSAIDs and postoperative use of local anesthetics.

a Multivariate linear regression model adjusted for age, sex, comorbidities, preoperative chronic pain, operation time, location of surgery, anesthesia methods, intraoperative consumption of opioids, intraoperative use of NSAIDs, postoperative use of NSAIDs and postoperative use of local anesthetics.

Adjusted *P*-Value was obtained via Holm’s procedure.

Severe pain: worst pain score≥7.

DEX, dexmedetomidine; IQR, interquartile range; NSAID, nonsteroidal anti-inflammatory drug; OR, odds ratio.

For continuous secondary outcomes, intraoperative DEX was associated with a reduction in the percentage of time spent in severe pain, the least pain score, and postoperative opioid consumption both in the univariate and multivariate linear regression analyses (Table 2; Supplementary 2, Table S2, Supplemental Digital Content 3, http://links.lww.com/JS9/A228). The correlation between intraoperative DEX and the worst pain scores was statistically significant only in univariate analysis (Supplementary 2, Table S2, Supplemental Digital Content 3, http://links.lww.com/JS9/A228).

For dichotomous outcomes, intraoperative use of DEX was associated with a reduction in postoperative anxiety, helplessness, nausea, drowsiness, pruritus, and patient’s wish for more treatment both in univariate and multivariate logistic regression analyses (Table 2; Supplementary 2, Table S2, Supplemental Digital Content 3, http://links.lww.com/JS9/A228). The detailed data of the multivariable regression analysis for the intraoperative use of DEX with postoperative patient satisfaction, percentage of time spent in severe pain, anxiety, and postoperative consumption of opioids are displayed in Supplementary 2, Table S3, Supplemental Digital Content 3, http://links.lww.com/JS9/A228. The receiver operating characteristic curves showed that the intraoperative use of DEX significantly improved the goodness of the fit of the regression models (Fig S1, Supplemental Digital Content 4, http://links.lww.com/JS9/A229).

### Propensity score-matched analysis

**Figure 2:**
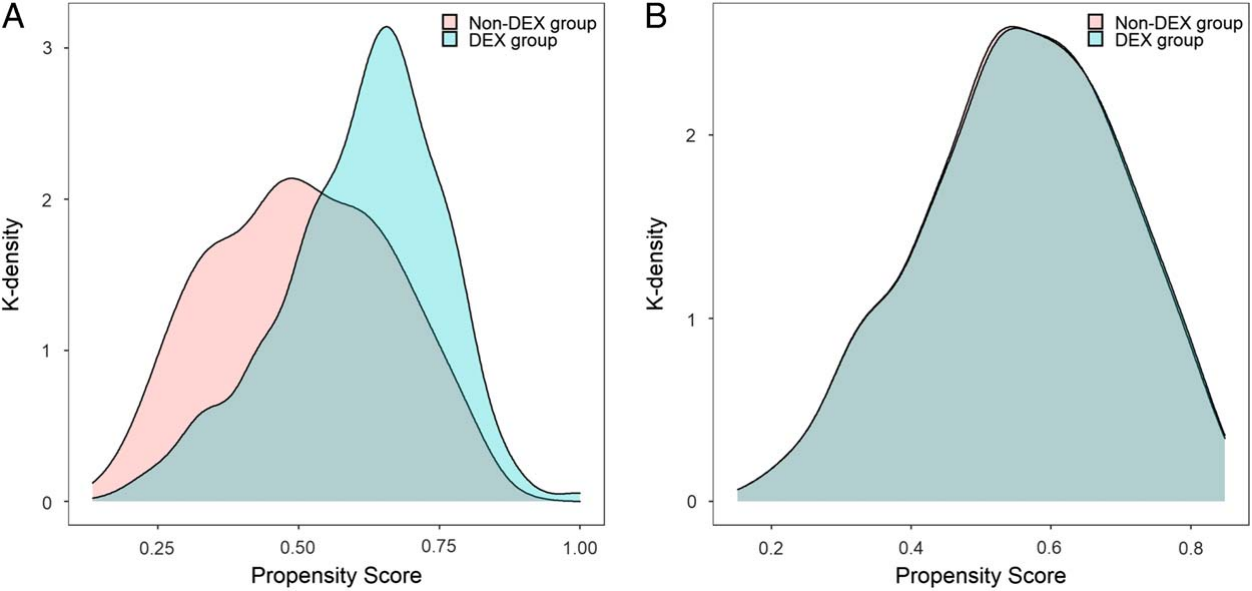
Distribution of propensity scores in the DEX group and non-DEX group. (A) Before matching. (B) After matching. DEX, dexmedetomidine.

We performed PSM analysis to further assess the value of intraoperative DEX on acute postoperative pain. Prior to matching, the median propensity score in patients in the DEX group was 0.589 (IQR: 0.512–0.663) versus 0.529 (IQR: 0.452–0.608) in those in the non-DEX group. PSM resulted in 415 patients in each group. The distribution of propensity scores in the two groups is graphically illustrated by kernel density estimation before and after matching (Fig. 2). After matching, the median propensity score was similar between those in the DEX group (0.560 [IQR: 0.458 – 0.663]) and the non-DEX group (0.561 [IQR: 0.458 – 0.660]). The baseline demographic and clinical characteristics of the variables were generally well balanced between the two groups, with standardized mean differences less than 0.10 for all covariates listed in Table 1. In the multivariate regression model after PSM, intraoperative use of DEX was associated with an improvement in patient satisfaction, and a reduction in the percentage of time in severe pain, the least pain score, postoperative opioid consumption, anxiety, helplessness, nausea, drowsiness, pruritus, and patients’ wish for more treatment (Table 2).

### Subgroup analysis

**Figure 3:**
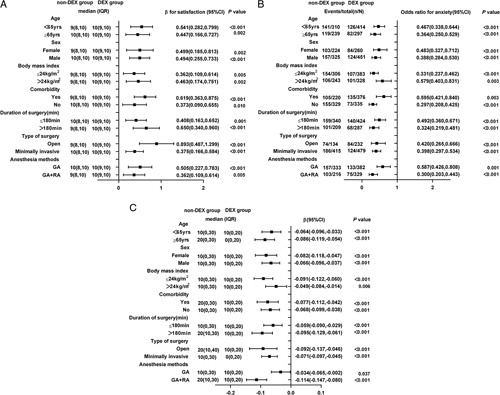
Forest plot of the subgroup analysis of the effect of intraoperative DEX on (A) patient satisfaction (β and adjusted 95% CIs are shown), (B) postoperative anxiety (odds ratio and adjusted 95% CIs are shown), and (C) percentage of time spent in severe pain (β and adjusted 95% CIs are shown) based on the original cohort. DEX, dexmedetomidine; GA, general anesthesia; RA, regional anesthesia.

Among the 711 patients in the DEX group, 297 (41.7%) were aged greater than or equal to 65 years, 260 (36.5%) were female, 328 (46.1%) had a BMI greater than 24 kg/m^2^, 376 (52.8%) presented with comorbidities, 287 (40.3%) had a surgery longer than 3 h, 479 (67.3%) underwent minimally invasive surgery, and 329 (46.2%) received GA combined with RA. Subgroup analyses for the intraoperative use of DEX on patient satisfaction (Fig. 3A), pain interference in anxiety (Fig. 3B), and percentage of time in severe pain (Fig. 3C) were summarized according to the above parameters. The use of intraoperative DEX was significantly associated with increased patient satisfaction and a reduction in pain interference in anxiety and the percentage of time in severe pain in all subgroups.

The dose of intraoperative DEX was recorded for 635 patients out of the 711 patients in the DEX group. The median dosage of DEX was 80 μg (IQR: 40–132 μg), that is, 1.2 μg/kg (IQR: 0.6–2.1 μg/kg). Our exploratory analysis of these 635 patients showed that the percentage of time spent in severe pain decreased with increasing intraoperative DEX dose. However, the dose of intraoperative DEX was not associated with patient satisfaction, anxiety, or postoperative opioid consumption (Supplementary 2, Table S4, Supplemental Digital Content 3, http://links.lww.com/JS9/A228).

## Discussion

In this study, we analyzed the association between the intraoperative use of DEX and the prognosis of acute postoperative pain in patients undergoing major gastrointestinal surgery using prospective data obtained from CAPOPS. We found that the intraoperative use of DEX was associated with the improvement of patient satisfaction and multiple pain-related outcomes, including the duration of severe pain, postoperative anxiety and helplessness, and postoperative opioid consumption.

As a crucial component of the multimodal analgesia approach, DEX has been widely used perioperatively for adjuvant sedation and analgesia in the clinical context^8^,^10^,^29^–^32^. In our analysis, the intraoperative use of DEX in patients undergoing major gastrointestinal surgery was as high as 56.4%, which indicated the wide acceptance of DEX for intraoperative use in China. However, in the international data we obtained from the PAIN-OUT registry, we have not observed such a high rate of DEX use (Supplementary 2, Table S5, Supplemental Digital Content 3, http://links.lww.com/JS9/A228). Therefore, further research is necessary to help clinicians evaluate the benefit of DEX administration regarding patient outcomes. In this cohort study, we used data from CAPOPS, which was the first nationwide registry focusing on acute postoperative pain in China. With previous experience from the PAIN-OUT project, we developed the CAPOPS registry. Strict training and a data safety monitoring system have assured the high quality of the data collected. In our analysis, we used multiple regression analysis and PSM to reduce the risk of confounding factors. However, there always remains a risk of residual or unmeasured confounders in cohort studies. In our primary analysis, more patients in the DEX group received RA than in the non-DEX group. Moreover, DEX was administered in patients with more comorbidities, those having more invasive procedures, and longer surgery duration. With PSM, it would appear that these covariates are balanced between the two groups. However, there are likely to be unmeasured or unmeasurable covariates that remain unbalanced. For example, there were multiple different techniques used in RA, which could not be balanced in our analysis. Therefore, our results should be confirmed by more definitive studies such as adequately sized randomized controlled trials.

In our analysis, intraoperative DEX did not decrease the incidence of severe pain or the worst pain experienced score in the first 24 h after surgery. These results are similar to those of a previous clinical trial^13^. However, we noted that the percentage of time spent in severe pain was significantly reduced in the DEX group compared to the non-DEX group. Our results show that intraoperative DEX may benefit these patients, mainly by decreasing the duration of severe pain. Our results also indicated that attention must be paid to the multiple aspects of postoperative pain, and pain scores should not serve as the only index to evaluate pain outcome.

Perioperative anxiety and helplessness are common in patients who undergo surgery and are related to negative behavioral manifestations and postoperative pain^33^. DEX can reduce postoperative anxiety and relieve postoperative restlessness, agitation, and pain in adult and pediatric patients^34^–^36^. However, Corbett *et al*.^37^ did not show the effectiveness of DEX in relieving postoperative anxiety. In this study, we evaluated the impact of pain on patients’ anxiety and helplessness using the IPOQ questionnaire rather than other measures of anxiety, such as the Hamilton Anxiety Rating Scale. From our results, postoperative pain caused different degrees of interference in patients’ anxiety and helplessness. A significantly lower rate of anxiety was reported in patients who received intraoperative DEX (29.3% in the DEX group and 47.4% in the non-DEX group). According to the concept of Minimal Clinically Important Difference from previous researchers^38^–^40^, the reduction in the incidence of anxiety was considered of clinical relevance. This finding could strengthen the rationale to use DEX in patients at high risk of postoperative anxiety.

Infusion of DEX during and after surgery improves subjective and objective sleep quality^41^. However, the timing and dosing of DEX administration have different effects on postoperative sleep quality. Patients who receive DEX in the daytime may have better improvements in sleep quality^42^. In addition, low-dose DEX may be an optimal treatment for postoperative sleep disturbances compared to high-dose DEX^43^,^44^. In our study, the variability of the timing and dosage of DEX in our patients may have contributed to the lack of differences in sleep quality between the two groups.

Intraoperative DEX is related to reduced postoperative opioid consumption^10^. Our result showed that postoperative opioid consumption was lower in the DEX group than that in the non-DEX group, which is consistent with the previous research^10^. At the same time, intraoperative DEX has also been associated with a reduction in opioid-related side effects, including nausea, dizziness, and pruritus. The reduction of postoperative opioid consumption possibly relates to the analgesic effect of DEX and may also be related to its haemodynamic effects.

This study had some limitations. First, we only included patients from China in our analysis. Certain pain management strategies in our patients may be quite different from those of other countries. For example, the proportion of patients with epidural or spinal analgesia in our study population was relatively low (11/545, 2.0%). We believe that the decreasing proportion of neuraxial analgesia is related to the popularization of nerve block techniques and minimally invasive surgery techniques. However, these differences need to be noted when extrapolating the results to international patients. In our exploratory analysis of international data obtained from the PAIN-OUT project, we obtained similar results (Supplementary 2, Table S5, Table S2, Supplemental Digital Content 3, http://links.lww.com/JS9/A228). However, the appendix results may be biased, as DEX use is not mandated to be recorded in the PAIN-OUT registry. Second, we only collected pain-related prognostic measures in the first 24 h after surgery and did not analyze the longer-term prognosis. Further study on the long-term outcomes of intraoperative DEX is necessary. Third, due to the observational design of the study, we did not have a standardized dosing regimen for DEX administration, nor did we analyze the relationship between the dose or timing of DEX administration and acute postoperative pain outcomes. This could be an important confounding factor in this study. Our exploratory analysis indicated that the dose of intraoperative DEX may be related to the duration of severe pain after surgery. However, from our study, no recommendation can be made on the dosage or mode of intraoperative DEX use.

## Conclusions

From the data analysis of a multicentre cohort, we conclude that intraoperative DEX use is associated with an improved prognosis of acute postoperative pain in multiple aspects in patients undergoing major gastrointestinal surgery, including increased patient satisfaction as well as decreased duration of severe pain, postoperative anxiety, helplessness, and postoperative opioid consumption. Future studies to determine the dose and timing of DEX administration on pain-related outcomes are warranted.

## Ethical approval

Ethical approval was innitially obtained from Chinese PLA General Hospital (S2017-033-02) and then aproved at each participating center. Written or oral consent was obtained from each participant according to the requirements of the local ethics committee.

## Sources of funding

This work was supported by the National Key Research and Development Program of China (No. 2018YFC2001905).

## Author contribution

L.X.: methodology, software, data curation, writing, original draft preparation, reviewing, and editing; Z.H.: methodology, software, visualization, formal analysis, writing, reviewing, and editing; G.J.: data curation, investigation, writing, reviewing, and editing; H.A.: software, validation, investigation, writing, reviewing, and editing. M.Y.: software, validation, writing, reviewing, and editing. Z.Z.: investigation, writing, reviewing, and editing. M.W.: conceptualization, supervision, writing, reviewing, and editing; Z.H.: conceptualization, supervision, writing, reviewing, and editing. L.Y.: conceptualization, methodology, writing, original draft preparation, writing - reviewing, and editing.

## Conflicts of interest disclosure

The authors declare that they have no conflict of interest.

## Research registration unique identifying number (UIN)

1. Name of the registry: Postoperative acute pain investigation
2. Unique Identifying number or registration ID: ChiCTR1900025237
3. Hyperlink to our specific registration : www.chictr.org.cn/hvshowproject.aspx?id=95278

## Guarantor

Liu Yanhong.

## Data availability statement

The data that support the findings of this study are available from the corresponding author upon reasonable request.

## Provenance and peer review

Not commissioned, externally peer-reviewed.

## Supplementary Material
