# Supplementary material for: Intraoperative dexmedetomidine on postoperative pain in gastrointestinal surgery: an observational study
Source: Int J Surg. 2023 Mar 31;109(4):887–95. doi: 10.1097/JS9.0000000000000360 (PMC10389438; doi:10.1097/JS9.0000000000000360)
Supplement: Supplementary file 2 [file js9-109-0887-s002.pdf]

# PATIENT OUTCOMES QUESTIONNAIRE

**Dear Sir \ Madam**

We would be grateful if you would participate in our survey on how patients feel after surgery. The aim of the survey is to improve the management of pain after surgery in this department.

Your participation is voluntary and the information you provide will be made anonymous once you hand in this questionnaire. This means that your name or other form of identification will be deleted from the questionnaire after you hand it in and will not be included in any records we will hold.

Your answers in this questionnaire will **not** be shared with your medical or nursing team.

Your team will treat you in the same way whether or not you choose to participate in our survey.

Many thanks for considering to take part in this survey.

PATIENT CODE:

# PATIENT OUTCOMES QUESTIONNAIRE

**The following questions are about pain you experienced since your surgery.**

**P1.** On this scale, please indicate the **worst pain** you had since your surgery:

| 0 | 1 | 2 | 3 | 4 | 5 | 6 | 7 | 8 | 9 | 10 |
|---|---|---|---|---|---|---|---|---|---|---|

**no pain** **worst pain possible**

**P2.** On this scale, please indicate the **least pain** you had since your surgery:

| 0 | 1 | 2 | 3 | 4 | 5 | 6 | 7 | 8 | 9 | 10 |
|---|---|---|---|---|---|---|---|---|---|---|

**no pain** **worst pain possible**

**P3.** How often were you in **severe pain** since your surgery?
Please circle your best estimate of the percentage of time you experienced **severe pain**:

| 0% | 10% | 20% | 30% | 40% | 50% | 60% | 70% | 80% | 90% | 100% |
|---|---|---|---|---|---|---|---|---|---|---|

**never in severe pain** **always in severe pain**

**P4.** Circle the one number below that best describes how much, since your surgery, **pain interfered with or prevented you from** ...

a. doing **activities in bed** such as turning, sitting up, changing position:

| 0 | 1 | 2 | 3 | 4 | 5 | 6 | 7 | 8 | 9 | 10 |
|---|---|---|---|---|---|---|---|---|---|---|

**did not interfere** **completely interfered**

b. **breathing deeply** or **coughing**:

| 0 | 1 | 2 | 3 | 4 | 5 | 6 | 7 | 8 | 9 | 10 |
|---|---|---|---|---|---|---|---|---|---|---|

**did not interfere** **completely interfered**

c. **sleeping**:

| 0 | 1 | 2 | 3 | 4 | 5 | 6 | 7 | 8 | 9 | 10 |
|---|---|---|---|---|---|---|---|---|---|---|

**did not interfere** **completely interfered**

d. Have you been **out of bed** since your surgery?

☐ Yes ☐ No

If yes, how much did **pain interfere or prevent you from doing activities out of bed** such as walking, sitting in a chair, standing at the sink:

| 0 | 1 | 2 | 3 | 4 | 5 | 6 | 7 | 8 | 9 | 10 |
|---|---|---|---|---|---|---|---|---|---|---|

**did not interfere** **completely interfered**

PATIENT CODE:

## PATIENT OUTCOMES QUESTIONNAIRE

**P5.** Pain can affect our mood and emotions.
On this scale, please circle the one number that best shows how much, since your surgery, **pain caused you to feel** ...

a. **anxious**

| 0 | 1 | 2 | 3 | 4 | 5 | 6 | 7 | 8 | 9 | 10 |
|---|---|---|---|---|---|---|---|---|---|---|

**not at all** **extremely**

b. **helpless**

| 0 | 1 | 2 | 3 | 4 | 5 | 6 | 7 | 8 | 9 | 10 |
|---|---|---|---|---|---|---|---|---|---|---|

**not at all** **extremely**

**P6.** Have you had any of the following **side effects** since your surgery?
Please circle "0" if no; if yes, circle the one number that best shows the severity of each:

a. **Nausea**

| 0 | 1 | 2 | 3 | 4 | 5 | 6 | 7 | 8 | 9 | 10 |
|---|---|---|---|---|---|---|---|---|---|---|

**none** **severe**

b. **Drowsiness**

| 0 | 1 | 2 | 3 | 4 | 5 | 6 | 7 | 8 | 9 | 10 |
|---|---|---|---|---|---|---|---|---|---|---|

**none** **severe**

c. **Itching**

| 0 | 1 | 2 | 3 | 4 | 5 | 6 | 7 | 8 | 9 | 10 |
|---|---|---|---|---|---|---|---|---|---|---|

**none** **severe**

d. **Dizziness**

| 0 | 1 | 2 | 3 | 4 | 5 | 6 | 7 | 8 | 9 | 10 |
|---|---|---|---|---|---|---|---|---|---|---|

**none** **severe**

**P7.** Since your surgery, how much **pain relief** have you received?
Please circle the one percentage that best shows how much relief you have received from all of your **pain treatments** combined (medicine and non-medicine treatments):

| 0% | 10% | 20% | 30% | 40% | 50% | 60% | 70% | 80% | 90% | 100% |
|---|---|---|---|---|---|---|---|---|---|---|

**no relief** **complete relief**

**P8.** Would you have liked **MORE pain treatment** than you received?

☐ Yes ☐ No

**P9.** Did you receive any **information** about your **pain treatment** options?

☐ Yes ☐ No

PATIENT CODE:

# PATIENT OUTCOMES QUESTIONNAIRE

**P10.** Were you **allowed to participate in decisions** about your **pain treatment** as much as you wanted to?

| 0 | 1 | 2 | 3 | 4 | 5 | 6 | 7 | 8 | 9 | 10 |
|---|---|---|---|---|---|---|---|---|---|---|

**not at all** **very much so**

**P11.** Circle the one number that best shows how **satisfied** you are with the results of your **pain treatment** since your surgery:

| 0 | 1 | 2 | 3 | 4 | 5 | 6 | 7 | 8 | 9 | 10 |
|---|---|---|---|---|---|---|---|---|---|---|

**extremely dissatisfied** **extremely satisfied**

**P12.** Did you use or receive any **non-medicine methods** to relieve your **pain**?

☐ Yes ☐ No

If yes, **check all** that apply:

☐ cold pack ☐ meditation ☐ deep breathing
☐ heat ☐ acupuncture ☐ prayer
☐ talking to medical staff ☐ walking ☐ massage
☐ talking to friends or relatives ☐ relaxation ☐ imagery or visualization
☐ TENS (Transcutaneous Electrical Nerve Stimulation)
☐ distraction (like watching TV, listening to music, reading)
☐ other (please describe):

**P13.** Did you have a **persistent painful condition for 3 months** or more before coming into hospital for this surgery?

☐ Yes ☐ No

a. If yes, **how severe** was the **pain** most of the time?
Please circle the number that indicates this.

| 0 | 1 | 2 | 3 | 4 | 5 | 6 | 7 | 8 | 9 | 10 |
|---|---|---|---|---|---|---|---|---|---|---|

**no pain** **worst pain possible**

b. If yes, **where** was this **persistent pain** located?

☐ site of surgery ☐ elsewhere ☐ both ( site of surgery and elsewhere )

## Thank you for your time and feedback

To be filled in by the research assistant Research assistant code:

Patient was interviewed: ☐ Yes ☐ No

If yes, please mark the reason(s):

☐ Too ill / weak ☐ Too much pain ☐ Requested assistance ☐ Did not understand scales
☐ Technical reasons (patient has no eyeglasses / is blind; can not sit up; is illiterate; arm is in cast; etc)

Version2.6 110225
