# Supplementary material for: Intraoperative dexmedetomidine on postoperative pain in gastrointestinal surgery: an observational study
Source: Int J Surg. 2023 Mar 31;109(4):887–95. doi: 10.1097/JS9.0000000000000360 (PMC10389438; doi:10.1097/JS9.0000000000000360)
Supplement: Supplementary file 3 [file js9-109-0887-s003.doc]

Supplemental Materials

Table S1 Specific local anaesthesia techniques

|  | Total (n=545) | Non-DEX group (n=216) | DEX group (n=329) |
| --- | --- | --- | --- |
| Local anaesthesia techniques, n(%) | 545(43.3) | 216(39.3) | 329(46.3) |
| Transversus abdominis plane block, TAP | 372(68.3) | 168(77.8) | 204(62.0) |
| Epidural | 8(1.5) | 5(2.3) | 3(0.9) |
| Spinal | 3(0.6) | 1(0.5) | 2(0.6) |
| Paravertebral | 27(5.0) | 7(3.2) | 20(6.1) |
| Quadratus lumborum block, QLB | 74(13.6) | 13(6.0) | 61(18.5) |
| Rectus sheath block, RSB | 7(1.3) | 2(0.9) | 5(1.5) |
| Erector spinae plane block, ESPB | 9(1.7) | 2(0.9) | 7(2.1) |
| Serratus anterior plane block, SAPB | 4(0.7) | 0(0.0) | 4(1.2) |
| Intercostal nerve block | 14(2.6) | 8(3.7) | 6(1.8) |
| Fascia iliaca compartment block, FICB | 1(0.2) | 0(0.0) | 1(0.3) |
| Wound infiltration, WI | 21(3.9) | 8(3.7) | 13(4.0) |
| TAP+WI | 2(0.4) | 0(0.0) | 2(0.6) |
| Paravertebral+WI | 1(0.2) | 1(0.5) | 0(0.0) |
| Others | 2(0.4) | 0(0.0) | 2(0.6) |

Table S2 Association between intraoperative DEX use and outcomes using univariate analysis

| **Outcomes** | **Univariate analysis** | |
| --- | --- | --- |
|  | **OR/β(95%CI)** | ***P*-Value** |
| **Primary outcome** |  |  |
| Satisfaction* | 0.476(0.289,0.662) | <0.001 |
| **Secondary outcomes** |  |  |
| Pain intensity |  |  |
| Worst pain score* | 0.330(0.100,0.559) | 0.005 |
| Least pain score* | -0.403(-0.544,-0.261) | <0.001 |
| Time spent in severe pain* | -0.063(-0.086,-0.041) | <0.001 |
| Severe pain# | 1.048(0.730,1.506) | 0.798 |
| Emotional impairment and ability to function |  |  |
| Anxiety# | 0.460(0.364,0.580) | <0.001 |
| Helplessness# | 0.601(0.464,0.779) | <0.001 |
| Moving in bed# | 0.850(0.579,1.248) | 0.407 |
| Deep breathing/coughing# | 0.632(0.464,0.862) | 0.004 |
| Sleep# | 1.006(0.805,1.257) | 0.957 |
| Postoperative opioid and adverse events |  |  |
| Postoperative consumption of opioids* | -11.582(-22.288,-0.875) | 0.034 |
| Nausea# | 0.554(0.441,0.696) | <0.001 |
| Drowsiness# | 0.539(0.423,0.688) | <0.001 |
| Pruritus# | 0.460(0.295,0.718) | 0.001 |
| Dizziness# | 0.766(0.608,0.966) | 0.024 |
| Perception of care |  |  |
| Wish for more treatment# | 0.417(0.324,0.538) | <0.001 |

Abbreviations: DEX, dexmedetomidine; OR, odds ratio; CI, confidence interval.

Severe pain: worst pain score ≥ 7.

# using logistic regression analysis; * using linear regression analysis.

Table S3 Multivariate analysis for satisfaction, time spent in severe pain, anxiety, and postoperative consumption of opioids in the original cohort.

| **Parameter** | **Satisfaction*** | | **Time spent in severe pain*** | | **Anxiety#** | | **Postoperative consumption of opioid*** | |
| --- | --- | --- | --- | --- | --- | --- | --- | --- |
|  | **β(95%CI)** | ***P*-Value** | **β(95%CI)** | ***P*-Value** | **OR(95%CI)** | ***P*-Value** | **β(95%CI)** | ***P*-Value** |
| **Age** | 0.008(0.000,0.016) | 0.056 | -0.002(-0.003,-0.001) | 0.001 | 0.990(0.980,1.001) | 0.07 | -0.600(-1.066,-0.134) | 0.012 |
| **Sex (male)** | 0.053(-0.138,0.243) | 0.587 | -0.005(-0.028,0.018) | 0.67 | 0.881(0.688,1.129) | 0.317 | 4.877(-5.909,15.662) | 0.375 |
| **Comorbidity** | -0.164(-0.353,0.024) | 0.088 | 0.034(0.012,0.057) | 0.003 | 1.469(1.147,1.881) | 0.002 | 4.385(-5.909,15.662) | 0.406 |
| **Preoperative chronic pain** | -0.277(-0.727,0.173) | 0.228 | 0.066(0.012,0.120) | 0.017 | 1.164(0.656,2.065) | 0.603 | -25.002(-47.121,-3.047) | 0.026 |
| **Intraoperative consumption of opioids** | 0.000(0.000,0.001) | 0.447 | 0.000(0.000,0.000) | 0.001 | 1.002(1.001,1.003) | 0.001 | -0.095(-0.152,-0.038) | 0.001 |
| **Intraoperative NSAIDs** | 0.560(0.368,0.752) | <0.001 | -0.047(-0.070,-0.024) | <0.001 | 0.699(0.543,0.900) | 0.005 | 10.086(-0.917,21.090) | 0.072 |
| **Postoperative NSAIDs** | 0.168(-0.145,0.481) | 0.292 | -0.008(-0.046,0.030) | 0.676 | 1.122(0.754,1.670) | 0.571 | -13.867(-26.262,-1.472) | 0.028 |
| **Postoperative local anaesthetics** | -0.090(-1.419,1.237) | 0.894 | -0.034(-0.194,0.126) | 0.677 | 0.717(0.122,4.207) | 0.713 | 13.924(-37.997,65.845) | 0.598 |
| **Duration of surgery** | -0.001(-0.003,0.000) | 0.049 | -0.000(0.000,0.000) | 0.506 | 0.997(0.996,0.999) | 0.008 | 0.044(-0.027,0.116) | 0.224 |
| **Location of surgery (Enterectomy)** | -0.124(-0.315,0.068) | 0.205 | -0.016(-0.040,0.007) | 0.161 | 0.741(0.577,0.952) | 0.019 | -20.740(-31.735,-9.746) | <0.001 |
| **Anaesthesia methods (GA+RA)** | -0.066(-0.267,0.134) | 0.516 | 0.038(0.014,0.062) | 0.002 | 0.995(0.765,1.293) | 0.967 | -5.735(-17.163,5.693) | 0.325 |
| **Intraoperative use of DEX** | 0.556(0.366,0.745) | < 0.001 | -0.081(-0.104,-0.058) | < 0.001 | 0.394(0.307,0.506) | < 0.001 | -16.342(-27.528,-5.155) | 0.004 |

Abbreviations: DEX, dexmedetomidine; OR, odds ratio; CI, confidence interval; NSAID, nonsteroidal anti-inflammatory drug; GA, general anaesthesia; RA, regional anaesthesia.

# using multivariate logistic regression analysis; * using multivariate linear regression analysis.

Table S4 Association between intraoperative DEX dose and satisfaction, time spent in severe pain, anxiety, and postoperative consumption of opioids using multivariate analysis.

| **Outcomes** | **OR/β(95%CI)** | ***P*-Value** |
| --- | --- | --- |
| Satisfaction* | 0.000197(-0.000618,0.001012) | 0.636 |
| Time spent in severe pain* | -0.000113(-0.000225,-0.000002) | 0.046 |
| Anxiety# | 0.998889(0.997348,1.000432) | 0.158 |
| Postoperative consumption of opioids* | -0.010519(-0.073034,-0.051996) | 0.741 |

Abbreviations: DEX, dexmedetomidine; OR, odds ratio; CI, confidence interval; NSAID, nonsteroidal anti-inflammatory drug.

#Multivariate logistic model adjusted for age, sex, comorbidities, preoperative chronic pain, operation time, location of surgery, anaesthesia methods, intraoperative consumption of opioids, intraoperative use of NSAIDs, postoperative use of NSAIDs and postoperative use of local anaesthetics.

*Multivariate linear regression model adjusted for age, sex, comorbidities, preoperative chronic pain, operation time, location of surgery, anaesthesia methods, intraoperative consumption of opioids, intraoperative use of NSAIDs, postoperative use of NSAIDs and postoperative use of local anaesthetics.

Table S5 Comparison of outcomes in the PAIN-OUT datasets.

| Outcomes | Non-DEX group, (n= 2506) | DEX group, (n=514) | OR/β(95%CI) | *P*-Value |
| --- | --- | --- | --- | --- |
| **Primary outcome** |  |  |  |  |
| Satisfaction, median(IQR)* | 9(7 - 10) | 8(7 - 10) | -0.394(-0.580,-0.207) | <0.001 |
| **Secondary outcomes** |  |  |  |  |
| **Pain intensity** |  |  |  |  |
| Worst pain score, median(IQR)* | 5(4 - 8) | 5(3 -7) | -0.235(-0.474,0.004) | 0.054 |
| Least pain score, median(IQR)* | 2(0 - 3) | 1(0 -3) | -0.212(-0.383,-0.042) | 0.015 |
| Time spent in severe pain, %, median(IQR)* | 30(10 - 50) | 20(0 - 40) | -0.065(-0.088,-0.042) | <0.001 |
| Severe pain , n (%)# | 972(38.8) | 164(31.9) | 0.713(0.577,0.881) | 0.002 |
| Emotional impairment and ability to function |  |  |  |  |
| Anxiety, n (%)# | 1383(55.2) | 253(49.2) | 0.737(0.604,0.899) | 0.003 |
| Helplessness, n (%)# | 1272(50.8) | 232(45.1) | 0.766(0.628,0.935) | 0.009 |
| Moving in bed, n (%)# | 2223(88.7) | 494(96.1) | 2.970(1.860,4.741) | <0.001 |
| Deep breathing/coughing, n (%)# | 2036(81.2) | 470(91.4) | 2.287(1.645,3.180) | <0.001 |
| Sleep, n (%)# | 1630(65.0) | 311(60.5) | 0.760(0.620,0.931) | 0.008 |
| Postoperative opioid and adverse events |  |  |  |  |
| Postoperative consumption of opioids, mg, median(IQR)* | 3.0(0 - 27.1) | 1.4(0 - 24.0) | 0.139(0.106,0.172) | <0.001 |
| Nausea, n (%)# | 1327(53.0) | 278(54.1) | 0.938(0.766,1.148) | 0.533 |
| Drowsiness, n (%)# | 1758(90.1) | 194(37.7) | 0.236(0.192,0.291) | <0.001 |
| Pruritus, n (%)# | 524(20.9) | 45(8.8) | 0.348(0.251,0.483) | <0.001 |
| Dizziness, n (%)# | 1235(49.3) | 232(45.1) | 0.793(0.649,0.969) | 0.023 |
| Perception of care |  |  |  |  |
| Wish for more treatment, n (%)# | 1577(62.9) | 278(54.1) | 0.633(0.518,0.773) | <0.001 |

Data were obtained from the PAIN-OUT registry with the same inclusion and exclusion criteria as described in the main text. 3020 cases were analysed with multivariable logistic regression# and linear regression*, adjusted for age, sex, comorbidities, preoperative chronic pain, operation time, surgery location, and anaesthesia methods.

Severe pain: worst pain score ≥ 7.

Abbreviations: DEX, dexmedetomidine; OR, odds ratio; CI, confidence interval, IQR, interquartile range.
