# Supplementary figures and images for: Intraoperative dexmedetomidine on postoperative pain in gastrointestinal surgery: an observational study
Source: Int J Surg. 2023 Mar 31;109(4):887–95. doi: 10.1097/JS9.0000000000000360 (PMC10389438; doi:10.1097/JS9.0000000000000360)

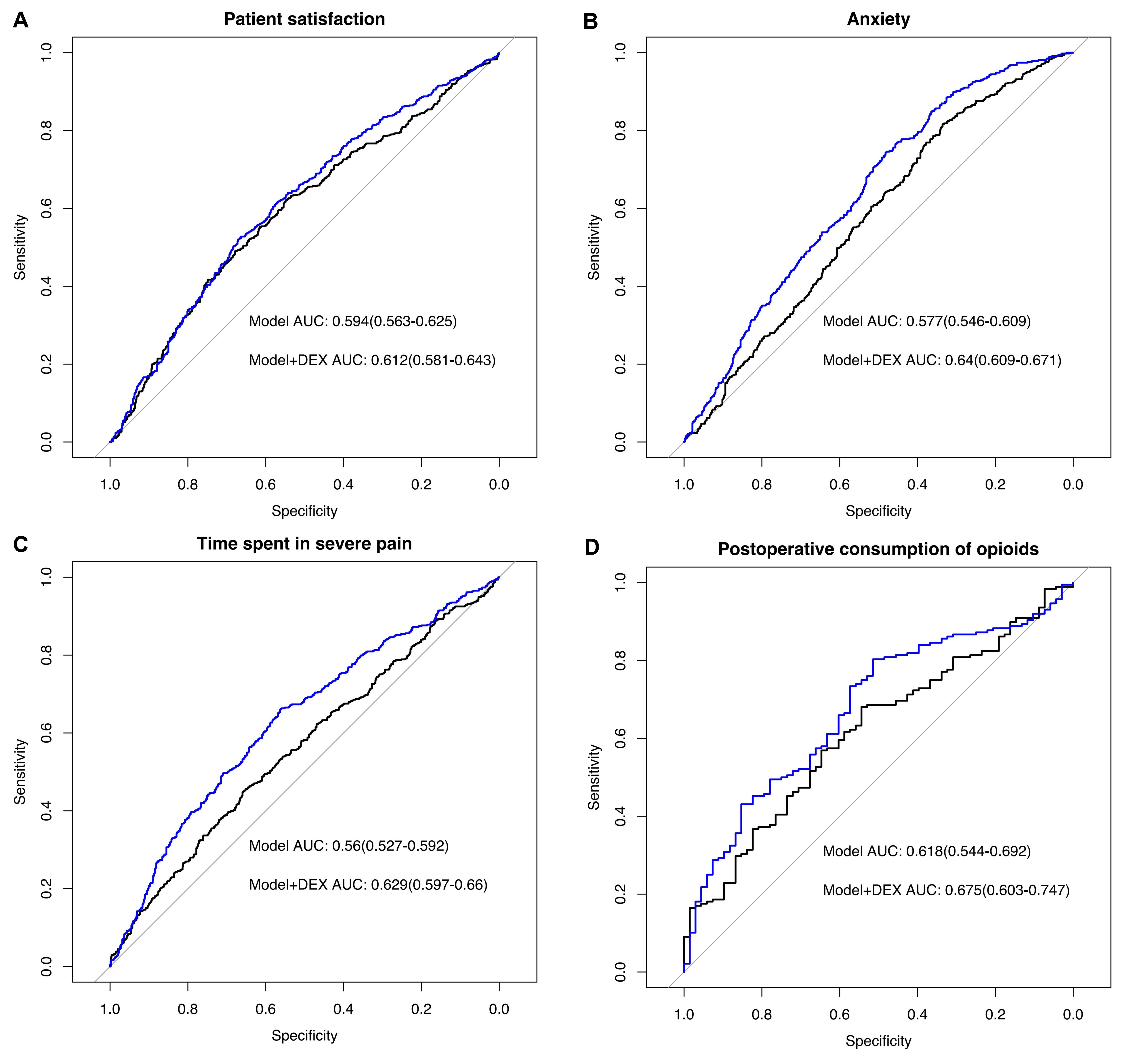

Supplement: Supplementary file 4 [file js9-109-0887-s004.jpg]
